# Corrosion Monitoring in Automotive Lap Joints Based on Imaging Methods of Lamb Waves

**DOI:** 10.3390/s24248092

**Published:** 2024-12-18

**Authors:** Yunmeng Ran, Cheng Qian, Xiangfen Wang, Weifang Zhang, Rongqiao Wang

**Affiliations:** 1 School of Energy and Power Engineering, Beihang University, Beijing 100191, China; rym2018@buaa.edu.cn (Y.R.);; 2 School of Reliability and Systems Engineering, Beihang University, Beijing 100191, China; cqian@buaa.edu.cn (C.Q.);

**Keywords:** corrosion monitoring, Lamb wave, automotive lap joint, piezoelectric sensor networks, damage imaging method

## Abstract

Corrosion damage presents significant challenges to the safety and reliability of connected vehicles. However, traditional non-destructive methods often fall short when applied to complex automotive structures, such as bolted lap joints. To address this limitation, this study introduces a novel corrosion monitoring approach using Lamb wave-based weighted fusion imaging methods. First, the Minimum Variance Distortionless Response (MVDR) is utilized to process Lamb wave signals collected under bolt-loosening and bolt-tightening conditions to image the bolt locations. Second, based on the identified bolt positions, the weighted Reconstruction Algorithm for Probabilistic Inspection of Damage (RAPID) is applied to the Lamb wave signals acquired before and after corrosion, enabling precise imaging of the actual positions of the corroded bolts. Experiments are conducted on three-bolt lap joints in cases of single-corrosion and two-corrosion using A0 mode Lamb waves and piezoelectric sensor networks. The results demonstrate that the proposed method effectively images multiple types of damage and achieves maximum location deviations of 7.43 mm. This approach enables precise and visual multi-damage assessment, particularly in hard-to-access regions. When integrated with V2X-enabled (Vehicle-to-Everything) systems, the method offers potential for incorporation into automotive structural health monitoring systems for remote diagnosis in complex structures, thereby enhancing monitoring efficiency and accuracy.

## 1. Introduction

In the automotive industry, corrosion damage is particularly critical in complex and inaccessible areas, where it can significantly compromise the strength and safety of the vehicle structure. Lap joints are commonly used in automotive manufacturing to connect metal parts, and these joints are often subjected to high mechanical stress and environmental exposure. Corrosion in these areas can weaken the joint, potentially leading to bolt failure, which, if not detected promptly, can result in substantial damage and costly repairs [1,2,3]. Consequently, monitoring corrosion in lap joints is essential for ensuring the ongoing reliability and safety of the vehicle. Traditional corrosion monitoring methods, such as visual inspection, are often time-consuming and impractical in inaccessible areas [4,5]. To address these challenges, structural health monitoring (SHM) techniques offer a more reliable and efficient solution. SHM involves the real-time assessment of a vehicle’s structure using sensor networks and monitoring systems, enabling continuous detection of corrosion and other structural damages. Non-destructive testing (NDT), an integral component of SHM, allows for the inspection of structural integrity without causing damage, making it widely applicable across various industries and scientific fields. This approach is particularly advantageous for detecting damage in complex structures, such as lap joints, that are difficult to inspect manually. Current NDT methods for corrosion monitoring include ultrasonic testing, eddy current testing, magnetic testing, radiographic testing, magnetic particle testing, etc. [6].

Among the above techniques, ultrasonic Lamb wave is a very promising one in the fields of SHM and NDT due to its superior capabilities, including long propagation distance, minimal attenuation, low detection cost, and high sensitivity to defects [7,8,9]. In recent years, Lamb waves have emerged as a promising tool for the detection of corrosion damage in metallic structures. The group velocity [10,11], multi-mode characteristics [12], nonlinear behavior [13] and wavefield patterns [14] have been extensively studied and utilized for corrosion diagnosis in plate-like structures. These methods are expected to be applicable to bolted lap joints. For example, the nonlinear characteristics of guided waves can be employed to assess corrosion levels in bolt heads using classifier algorithms [15]. Additionally, the effects of bolt loosening on ultrasonic Lamb waves are analogous to those of bolt corrosion. The propagation model of Lamb waves in loose bolts has been investigated, offering valuable insights into the propagation mechanisms of Lamb waves in corroded bolted structures [16,17].

Tomographic imaging methods utilizing ultrasonic Lamb waves for corrosion damage assessment have garnered considerable attention in the fields of SHM and NDT. Techniques for corrosion localization involve the use of piezoelectric sensors arranged in sparse sensor arrays, including delay-and-sum (DAS) imaging [18], spatial wavenumber imaging [19], minimum variance distortionless response (MVDR) imaging [20], algebraic reconstruction technique (ART) [21], and reconstruction algorithm for the probabilistic inspection of damage (RAPID) imaging [22,23]. Guided wave tomography (GWT) has also demonstrated significant potential for quantifying corrosion thickness and estimating remaining structural life [24,25]. These tomography methods are particularly noteworthy for their reliability and effectiveness in localizing damage over large structural areas, delivering accurate and high-resolution images [26,27,28]. Furthermore, the proposed methods offer advantages such as rapid computation and ease of implementation in complex structures, including bolted lap joints and rib-to-deck joints [20,29]. The above imaging methods enable remote real-time damage monitoring of inaccessible areas, allowing for timely acquisition of information about the health of the structure, thereby enhancing its reliability and service life. Therefore, it is reasonable to expect that these methods will be satisfactory in multi-corrosion imaging of lap joints.

To effectively address the challenge of monitoring corrosion in bolted lap joints, this paper employs an innovative non-destructive testing method, utilizing A0 mode Lamb waves and weighted fusion imaging algorithms to characterize the location of corrosion damage. This innovative method integrates the advantages of the MVDR algorithm for identifying bolt loosening and tightening states, along with the RAPID algorithm for damage imaging. This method comprises two steps: First, the MVDR imaging algorithm is utilized to process Lamb wave signals collected under both bolt-loosening and bolt-tightening conditions, thereby identifying the locations of the bolts within the monitored region. Second, based on the identified bolt positions, the weighted RAPID imaging algorithm is applied to the Lamb wave signals acquired before and after corrosion, enabling precise imaging of the actual positions of the corroded bolts. To validate the proposed approach, experiments are conducted on a three-bolt lap joint structure, including both single-corrosion and two-corrosion tests. The results demonstrate that the proposed method offers the highest imaging accuracy in comparison with the traditional RAPID algorithm, and achieves the maximum location deviations of 7.43 mm in two-corrosion imaging. Therefore, the proposed weighted fusion imaging method is more suitable for the visual assessment of multi-damage issues and can be applied to lap joints in areas that are inaccessible or difficult to observe directly. The findings of this study are also expected to be implemented with V2X (Vehicle-to-Everything) communication technology, to provide real-time data and proactive maintenance strategies. The proposed method offers potential for incorporation into automotive structural health monitoring systems for remote diagnosis in complex structures, thereby enhancing monitoring efficiency and accuracy.

The organization of this paper is as follows: In Section 2, the weighted fusion imaging method are briefly introduced. In Section 3, the experiments on corrosion monitoring in bolted lap joints based on Lamb wave are performed. In Section 4, the signal analysis and imaging results of single-corrosion and two-corrosion are presented. In Section 5, several conclusions are drawn.

## 2. Methodologies

### 2.1. Dispersion of Lamb Wave

Lamb waves are a category of ultrasonic waves which are guided between two parallel free surfaces. There are two primary modes of Lamb wave propagation in the plate: the symmetric mode (S) and the anti-symmetric mode (A). The symmetric mode comprises patterns such as S0, S1, …, Sn, etc., while the anti-symmetric mode comprises patterns such as A0, A1, …, An, etc. [30]. The propagation of Lamb waves is represented as a superposition of multiple dispersive wave modes. For each Lamb wave mode, its propagation velocity in an isotropic plate depends on the product of frequency and plate thickness.

**Figure 1:**
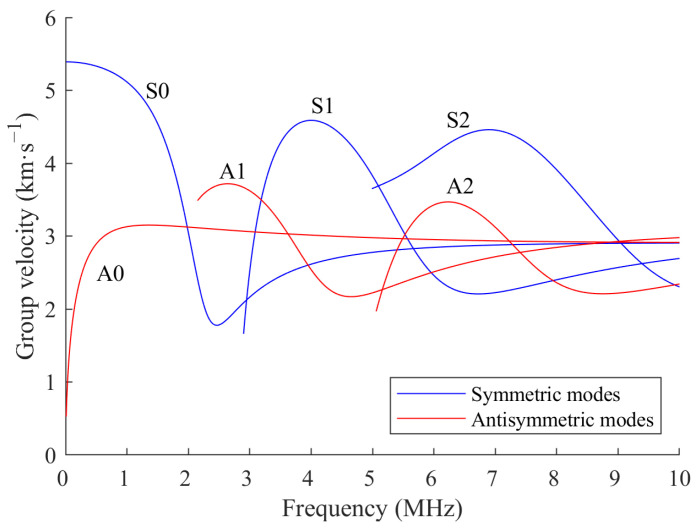
Dispersion curves of group velocity versus frequency for symmetric and antisymmetric modes of Lamb waves in an aluminum plate with 2 mm thickness.

The dispersion curves of group velocity, representing the velocity of the wave packet when Lamb waves propagate in a 2024-T3 aluminum plate with a thickness of 2 mm, are presented in Figure 1 [31]. The material properties of 2024-T3 aluminum are listed in Table 1. It can be seen that in the low-frequency range (i.e., <1 MHz), there are only S0 mode and A0 mode in the Lamb wave, which are the simplest and most fundamental modes of guided waves. The group velocity of these two modes within the low-frequency range differs significantly, making it relatively simple to separate the corresponding mode waveforms.

### 2.2. MVDR Imaging Method

**Figure 2:**
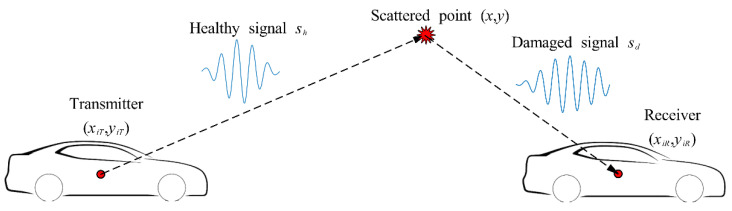
The propagation of a scattered signal with its transmitter and receiver sensors.

MVDR imaging calculates each pixel value individually based on differenced signals. Existing literature has shown that the MVDR algorithm can identify bolt looseness in lap-joint structures [20]. Therefore, signals before and after tightening bolts using a torque wrench can be utilized to localize bolt positions. As shown in Figure 2, the damage is assumed to be a scattered point at coordinates (*x*, *y*). The scattered signal is generated from the transmitter, reaches the damage, and is subsequently received by the receiver. The scattered signal is defined as the difference between the response signal in the damaged state and in the healthy state:

(1) $$r=s_{d}-s_{h}$$

It is assumed that there are *M* transmitter-receiver sensor pairs. At each pixel point (*x*, *y*), the amplitudes of all scattered signals are grouped as a vector:

(2) $$r(x,y)=\left[r_{1}(t_{1}(x,y)),r_{2}(t_{2}(x,y)),\cdots ,r_{i}(t_{i}(x,y)),\cdots ,r_{M}(t_{M}(x,y))\right]$$

where *r_i_*(*t*) is the scattered signal of *i*th transmitter-receiver pair. To enhance robustness and simplify practical implementation, the scattered signals presented in Equation (2) can be replaced with their Hilbert envelope. *t_i_*(*x*, *y*) is the time of flight (ToF) of the *i*th transmitter-receiver at position (*x*, *y*), which is defined as follows:

(3) $$t_{i}(x,y)=\frac{\sqrt{{\left(x-x_{iT}\right)}^{2}+{\left(y-y_{iT}\right)}^{2}}+\sqrt{{\left(x-x_{iR}\right)}^{2}+{\left(y-y_{iR}\right)}^{2}}}{c_{g}}$$

where (*x_iT_*, *y_iT_*) and (*x_iR_*, *y_iR_*) represent the transmitter and receiver positions of *i*th sensor pair, respectively.

In the traditional delay-and-sum method, the pixel value at (*x*, *y*) is calculated by the superposition of the scattered signal amplitudes from all transmitter-receiver pairs, which may lead to the formation of imaging artifacts in areas remote from the damage [32]. To suppress these artifacts, the pixel intensity at (*x*, *y*) in minimum variance imaging is calculated as [33]:

(4) $$P_{xy}=w_{xy}^{T}R_{xy}w_{xy}=w_{xy}^{T}(r_{xy}r_{xy}^{T})w_{xy}$$

where ***r****_xy_* is the amplitude vector in Equation (2), the superscript T denotes the transposition operation, and ***R****_xy_* is the correlation matrix of the amplitude vector ***r****_xy_*. ***w****_xy_* is a vector of weights which is obtained by solving the following constrained optimization problem:

(5) $$\min w_{xy}^{T}R_{xy}w_{xy}\;s.t.\;w_{xy}^{T}e_{xy}=1$$

where ***e****_xy_* is referred to as the look-direction. In this paper, ***e****_xy_* is simplified as $e_{xy}∼\left[1,1,\cdots ,1\right]$ [29].

The optimal solution to Equation (5) can be obtained by introducing a Lagrange multiplier and the closed-form solution is expressed as follows:

(6) $$w_{xy}=\frac{R_{xy}^{-1}e_{xy}}{e_{xy}^{T}R_{xy}^{-1}e_{xy}}$$

where $R_{xy}^{-1}$ is the inverse matrix of ***R****_xy_*. In order to avoid the ill-posed problem associated with matrix inversion, diagonal loading is typically employed in the regularization of the matrix inversion [34]:

(7) $$R_{xy}^{-1}={\left(R_{xy}+f\lambda_{1}I\right)}^{-1}$$

where *f* is the coefficient to control the degree of diagonal loading, *λ*_1_ is the largest eigenvalue of matrix ***R****_xy_*, and ***I*** is the identity matrix. Existing studies indicate that when *f* = 1, the imaging results closely resemble those of the DAS imaging method [29]. As *f* decreases, the suppression of imaging artifacts within the imaging region improves [32]. However, when *f* < 0.01, the imaging results exhibit no significant difference compared to those obtained at *f* = 0.01. Therefore, in this study, the diagonal loading coefficient *f* is set to 0.01.

Substituting the value from Equation (7) into Equation (6) leads to the vector ***w****_xy_* and the minimum pixel value *P_xy_* at coordinates (*x*, *y*). A larger value of *P_xy_* indicates a higher probability of damage at the pixel location (*x*, *y*). Consequently, bolt locations can be identified through an analysis of the imaging results.

### 2.3. Weighted RAPID Imaging Method

When the bolt position is known, it can be utilized as prior information for path weighting, thereby enhancing the accuracy of corrosion imaging. The weighted RAPID imaging method consists of three main steps: calculation of the Signal Difference Coefficient (SDC), path weighting and damage imaging. First, the changes in signal before and after damage are correlated with the differences between the undamaged and damaged states of the structure [35]. The SDC quantitatively evaluates changes in the Lamb wave signal between the undamaged and damaged conditions, and is defined by the following equation:

(8) $$SDC=1-\left|\frac{\int_{\Delta T}\left(s_{h}-{\bar{s}}_{h}\right)\left(s_{d}-{\bar{s}}_{d}\right)}{\sqrt{\int_{\Delta T}{\left(s_{h}-{\bar{s}}_{h}\right)}^{2}}\sqrt{\int_{\Delta T}{\left(s_{d}-{\bar{s}}_{d}\right)}^{2}}}\right|$$

where Δ*T* represents the time frame of the wave packet, *s_h_* and *s_d_* represent the Lamb signal in a healthy state and damaged state, respectively. ${\bar{s}}_{h}$ and ${\bar{s}}_{d}$ represent the average values of signal *s_h_* and *s_d_*, respectively. An elevated SDC value suggests a higher likelihood of damage along the signal path.

Secondly, the sensing path weight is calculated based on the prior bolt localization results. For paths that pass through the bolt imaging region, a weight of 1 is assigned. For paths that do not pass through the prior imaging region, the contribution of their SDC to the imaging results is reduced [36]. The weighting coefficient of path *i* is defined as follows:

(9) $$w_{i}=\left\{\begin{array}{c} 1,\;\;\;\;\;\mathrm{if}\;\mathrm{path}\;i\;\mathrm{passing}\;\mathrm{through}\;\mathrm{bolt}\\ 0.2,\;\;\mathrm{if}\;\mathrm{path}\;i\;\mathrm{not}\;\mathrm{passing}\;\mathrm{through}\;\mathrm{bolt} \end{array}\right.$$

**Figure 3:**
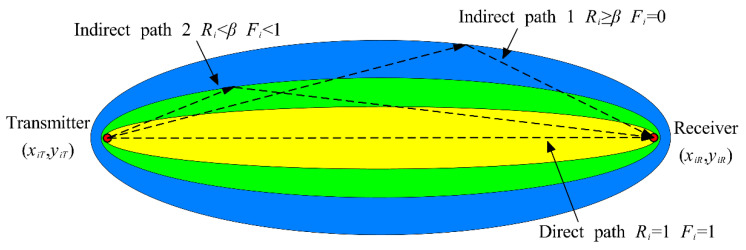
Illustration of the elliptical distribution function of the RAPID algorithm, with transmitter and receiver sensors at the foci.

Thirdly, the probability of damage occurrence at a specific point is determined by SDC, the weighting coefficient and the relative position to sensor pairs [37]. Essentially, the damage probability at a given coordinate (*x*, *y*) is influenced by each transmitter-receiver pair if the coordinate lies within its elliptical distribution. The elliptical distribution function for the *i*th transmitter-receiver pair, with the foci at the sensor locations, can be expressed as follows:

(10) $$\left\{\begin{array}{c} F_{i}(x,y)=\frac{\beta -R_{i}(x,y)}{\beta -1},\beta >R_{i}\\ F_{i}(x,y)=0,\;\beta \leq R_{i} \end{array}\right.$$

where *β* is the scaling parameter which controls the shape of the elliptical distribution. A decrease in *β* results in increased elongation of the ellipse [38]. In this study *β* is set to 1.05, as this value has been shown in existing literatures to be sufficient for producing accurately localized imaging results [39,40]. *R_i_*(*x*, *y*) represents the geometrical weight of the *i*th transmitter-receiver pair at coordinates (*x*, *y*), which is defined as follows:

(11) $$R_{i}(x,y)=\frac{\sqrt{{\left(x-x_{iT}\right)}^{2}+{\left(y-y_{iT}\right)}^{2}}+\sqrt{{\left(x-x_{iR}\right)}^{2}+{\left(y-y_{iR}\right)}^{2}}}{\sqrt{{\left(x_{iT}-x_{iR}\right)}^{2}+{\left(y_{iT}-y_{iR}\right)}^{2}}}$$

where (*x_iT_*, *y_iT_*) and (*x_iR_*, *y_iR_*) are the transmitter and receiver positions of *i*th sensor pair, respectively. The elliptical distribution established by the equations presented above in the RAPID algorithm is illustrated in Figure 3. It is evident that when damage occurs along the direct path, both the geometrical weight and elliptical distribution reach a value of 1. Conversely, if the damage is located away from the direct path of the sensor pair, both the geometrical weight *R_i_*(*x*, *y*) and elliptical distribution *F_i_*(*x*, *y*) associated with that sensor pair decrease. Therefore, the probability distribution of damage at coordinates (*x*, *y*) can be estimated as a linear summation of contributions from each transmitter-receiver pair, which can be expressed as follows:

(12) $$P(x,y)=\sum_{i=1}^{M}SDC\cdot F_{i}(x,y)$$

where *M* is the total number of sensors pairs. Therefore, the location of the damage is identified with a considerably higher probability than the remaining points. Finally, damage reconstruction images are generated by normalizing the probability *P*(*x*, *y*) between 0 and 1.

### 2.4. Weighted Fusion Bolt Corrosion Imaging Algorithm

**Figure 4:**
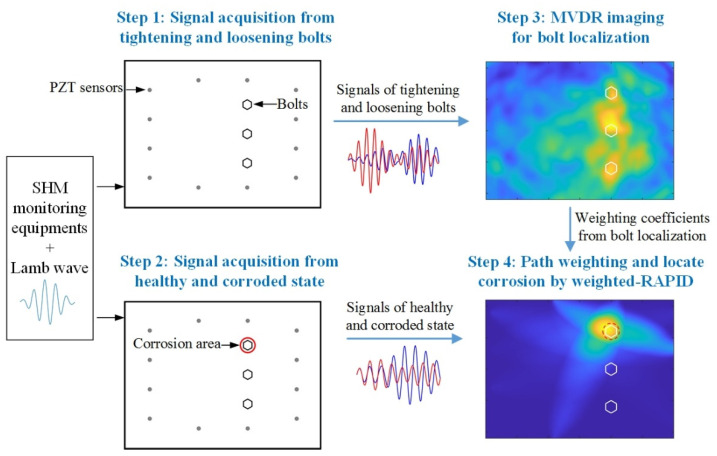
Framework of the weighted fusion imaging algorithm.

In lap-joint structures, bolts are the most susceptible to fatigue and corrosion due to the influence of stress. This paper proposes a weighted fusion corrosion imaging algorithm to enhance the localization performance of corroded bolts in lap-joint structures. It utilizes the bolt localization results as prior information to weight the sensing paths, then employs a weighted imaging method for accurate localization of corroded bolts. The steps of this method are illustrated in Figure 4 as follows:

**Step 1:** Set the experimental equipment and collect the signals before and after tightening bolts.
**Step 2:** Conduct the corrosion experiment and collect the signals before and after corrosion.
**Step 3:** Use the MVDR algorithm and the signals from Step 1 to image the bolt localization.
**Step 4:** Calculate the weighting coefficients based on the bolt imaging and use the weighted RAPID algorithm along with the signals from Step 2 to perform corroded bolts imaging.

## 3. Experiments of the Bolted Lap Joints

### 3.1. Experimental Equipment

**Figure 5:**
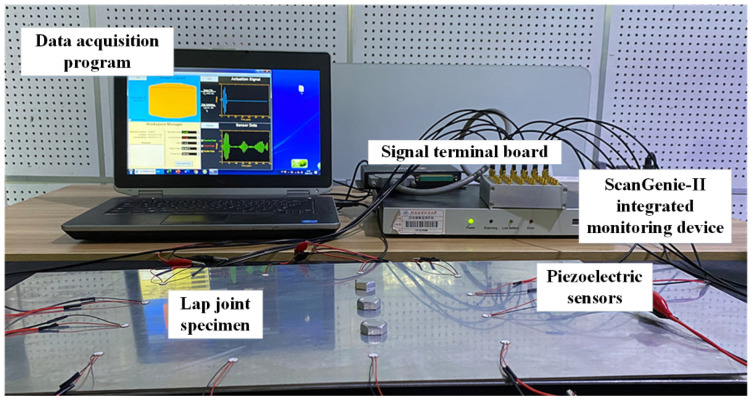
Structural health monitoring scanning system for the lap joints (the monitoring device including a signal generator, a power amplifier and a response channel scanning module).

In this study, the Lamb wave generation and acquisition processes are conducted using the ScanGenie-II integrated structural health monitoring scanning system, produced by Acellent Technologies (Sunnyvale, CA, USA). The monitoring system comprises a data acquisition program, an integrated monitoring device, a signal terminal board, and three-bolt lap joint specimens equipped with piezoelectric sensors, as illustrated in Figure 5. The monitoring device constitutes a hardware integrated system, including a signal generator module, a power amplifier and a response channel scanning module. Within the signal generator module, a sinusoidal modulation wave generates an excitation signal, which is amplified by the power amplifier before propagating through the structure. Finally, the Lamb wave signal is captured by the response channel scanning module using piezoelectric sensors at a sampling rate of 12 M/s. The piezoelectric sensors are manufactured by STEINER & MARTINS, INC. (39873 Highway 27, Suite 225, Davenport, FL, USA), and their performance is detailed in Table 2.

For this study, a five-cycle sinusoidal tone burst modulated by a Hanning window is selected as the excitation pulse due to its periodicity, smoothness and rapid peak time characteristics. The central frequency of the excitation signal is set within the range of 140–170 kHz to prevent issues such as wave overlap caused by long envelope periods, as well as excessive reflected and scattered waves caused by short envelope periods. The excitation signal is formulated as follows:

(13) $$u(t)=A\left(1-\cos\frac{2\pi f_{c}t}{N}\right)\sin2\pi f_{c}t$$

where *A* is the amplitude of the signal, *f_c_* is the center excitation frequency, *N* is the number of excitation signal cycles, and *H*(*t*) is the Heaviside step function.

### 3.2. Case I: Bolted Lap Joint with Single Corrosion

**Figure 6:**
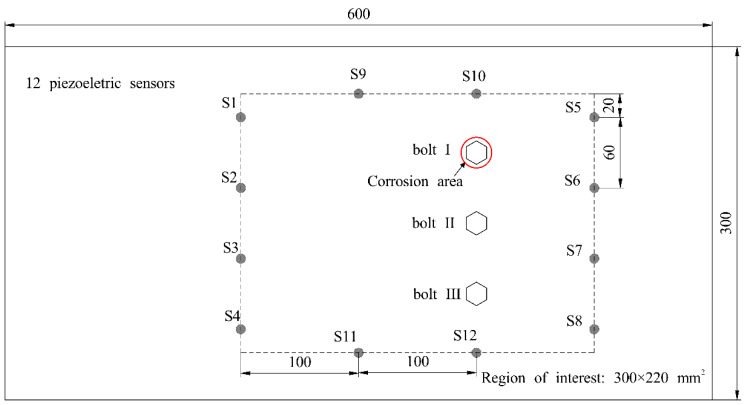
Schematic graph of a lap joint specimen with single corrosion and sensors array. The red circle denotes corrosion area.

The lap joint specimen consists of two identical aluminum plates connected by three fully threaded M12 bolts, as illustrated in Figure 6. Each aluminum plate measures 600 mm × 300 mm × 2 mm, and the material properties are given in Table 1. A static load of 30 Nm is applied to the bolts using a torque wrench. In Figure 6, a schematic of the lap joint specimen highlights the corrosion area around bolt I. A rectangular region of interest measuring 300 mm × 220 mm is surrounded by a sensor array containing 12 piezoelectric sensors and 52 effective monitoring paths. The parameters and geometry of the piezoelectric sensors are detailed in Table 2. Lamb wave signals are collected before and after the tightening of the three bolts to locate their positions. Subsequently, a corrosion test is conducted. After corrosion, Lamb wave signals are again collected for imaging and locating the corroded bolts.

**Figure 7:**
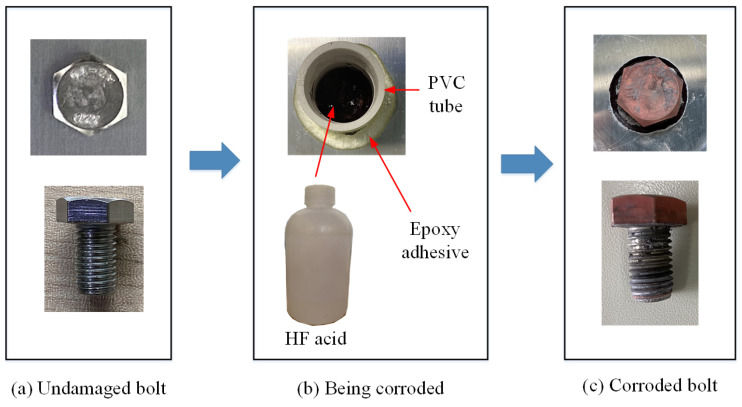
The corroding process in the single corrosion test on a bolted lap joint: (**a**) before corrosion; (**b**) being corroded by using HF acid; (**c**) after corrosion.

In the single corrosion test, bolt I corroded while bolts II and III remained undamaged. The initial state of undamaged bolt I is depicted in Figure 7a. Corrosion was induced using dilute hydrofluoric (HF) acid with a PVC tube of 26 mm internal diameter, as shown in Figure 7b. The PVC tube was affixed to the bolt position using a corrosion-resistant epoxy adhesive. Upon solidification of the adhesive, 6 mL of 10% HF acid was injected into the PVC tube, initiating corrosion of the aluminum plate and bolt. After nine hours, the cessation of bubbling in the PVC tube indicated the completion of the corrosion process. To extend the corrosion duration, the solution was removed from the PVC tube, then an equivalent volume and concentration of HF solution was reinjected. After a corrosion period exceeding ten hours, a slow, gradual flow of acid solution was observed on the screw. At the conclusion of the subsequent corrosion process, the PVC tube, corrosion by-products and adhesive were removed from the lap joint surface. The corroded state of the bolt is shown in Figure 7c, with a cumulative corrosion time of 18 h. It can be observed that a minimal amount of acid solution penetrated the through-hole during the corrosion process, leading to localized corrosion of the screw. As sufficient torque was applied to the bolts, the acid solution did not diffuse between the aluminum alloy plates or cause significant damage; instead, it flowed out along the screw.

### 3.3. Case II: Bolted Lap Joint with Two Corrosions

**Figure 8:**
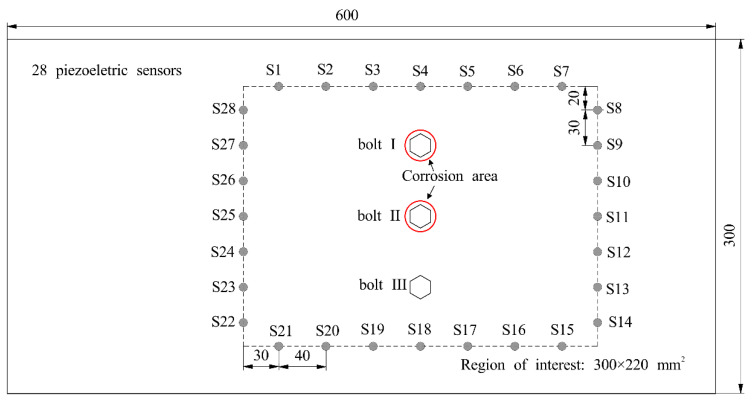
Schematic graph of a lap joint specimen with two corrosions and sensors array. The red circle denotes corrosion area.

The two-corrosion test is conducted on a lap joint with identical dimensions and material properties as described in Section 3.2. The region of interest, measuring 300 mm × 220 mm, is enclosed by a rectangular sensor array consisting of 28 PZT sensors and 294 effective monitoring paths, as shown in Figure 8. Figure 8 indicates that the area between the two corrosion bolts should be classified as undamaged, rather than being misunderstood as an artifact. Consequently, additional PZT sensors and sensing paths are employed in the test to capture signals from the paths that traverse the undamaged area. The experimental setup and excitation signal function remain consistent with those described in Section 3.2.

**Figure 9:**
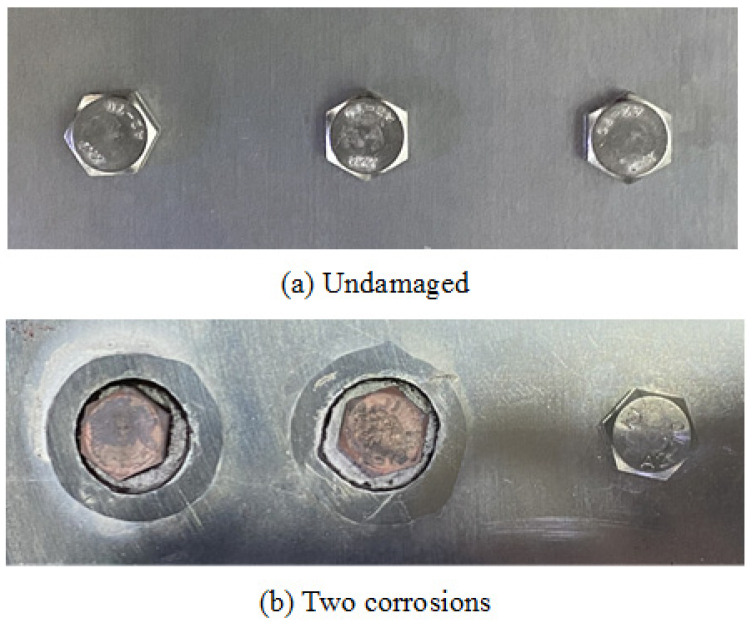
The bolted lap joint specimen with two corrosions: (**a**) before corrosion, (**b**) after corrosion.

In the two-corrosion test, both bolt I and bolt II underwent simultaneous corrosion, while bolt III remained intact. The states of the bolts before and after corrosion are illustrated in Figure 9. Two PVC tubes, each with an internal diameter of 26 mm, were affixed to the positions of bolt I and bolt II using epoxy adhesive. After the adhesive around PVC tubes solidified, 8 mL of 10% HF acid was injected into each tube. After ten hours, the acid solution in the PVC tubes ceased to corrode. Then the acid solution was removed from the tubes, and an equivalent volume and concentration of HF solution was reinjected. This procedure allowed for a cumulative corrosion time of 20 h for each bolt. The other corrosion procedures are consistent with those employed in the single corrosion test as described in Section 3.2.

## 4. Discussion

### 4.1. Imaging Results of Single Corrosion

Firstly, the response signals before and after bolt tightening are analyzed. A compromise frequency of 160 kHz is selected from the excitation frequency range for the subsequent signal analysis. Due to the complex structure of the lap joint and the presence of circular corrosion, the received signals typically contain direct wave packet of a specific mode, boundary reflection wave packet, and other wave packets resulting from complex boundary reflections or scattering. In the presence of these overlapping wave packets, identifying specific wave packets through envelope peaks may prove challenging. By calculating the arrival times of the direct wave packet and scattered wave packet from the group velocity in Figure 1, specific wave packets of specific modes can be extracted from the response signals.

**Figure 10:**
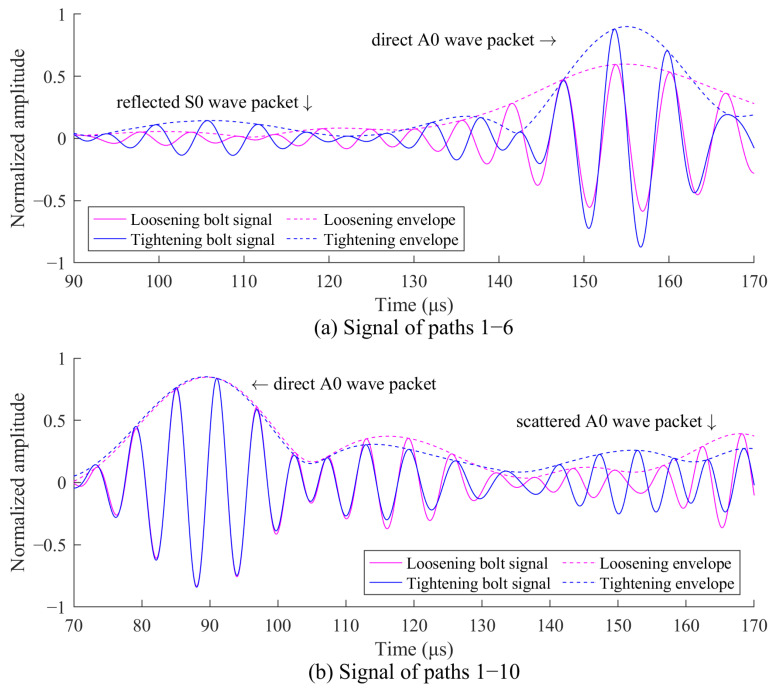
Response signals before and after bolt tightening in the single corrosion test: (**a**) paths 1–6; (**b**) paths 1–10.

Figure 10 shows the received Lamb waves along paths 1–6 through the bolts and paths 5–9 that do not pass through the bolts. Due to the experimental equipment, the direct S0 wave packet is obscured by crosstalk. In Figure 10a, the time range of 90–120 μs corresponds to the reflected S0 wave packet, and 140–170 μs corresponds to the direct A0 wave packet. It is evident that the signal amplitudes of both the S0 and A0 modes are influenced by bolt tightening, with the A0 mode showing more pronounced changes. In Figure 10b, the direct A0 wave packet amplitude in the range of 70–100 μs remains relatively unchanged, while the scattered A0 wave packet emerges as a result of bolt tightening. Previous studies have demonstrated that A0 mode Lamb waves exhibit higher energy and sensitivity to corrosion damage at relatively low frequencies, resulting in improved signal-to-noise ratios [35,41]. By analyzing the A0 mode wave within the received signals, it is possible to determine if a bolt is present along the signal path.

**Figure 11:**
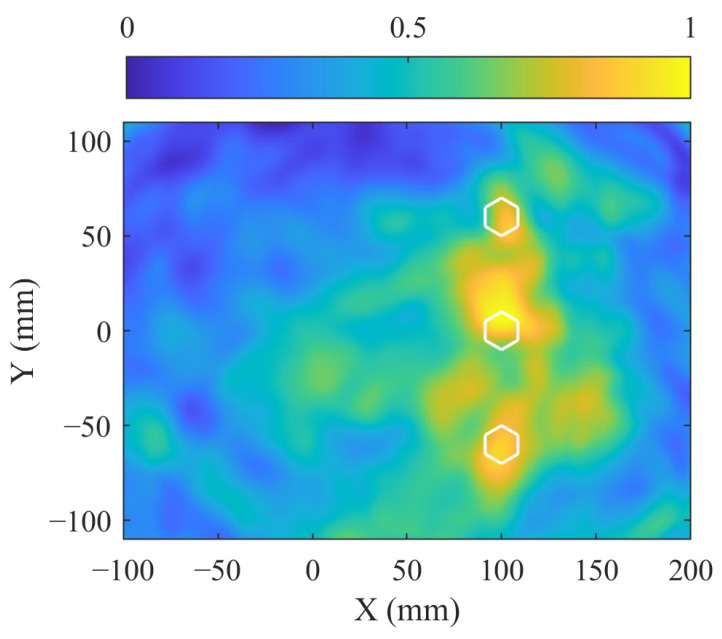
Imaging result of bolt localization using MVDR in the single corrosion test, with the white hexagons denoting the bolts.

Therefore, the signals before and after bolt tightening can be utilized to image the bolt locations. In Figure 11, the bolts are indicated by white hexagons. It is evident that the highlighted imaging area corresponds to the actual bolt positions, demonstrating the accurate characterization of the bolt’s loosening and tightening states by the MVDR algorithm. There are 21 sensing paths passing through the highlighted imaging region, and 31 sensing paths not passing through it. Based on the imaging result, a weighting coefficient for the 52 paths is determined using Equation (9).

**Figure 12:**
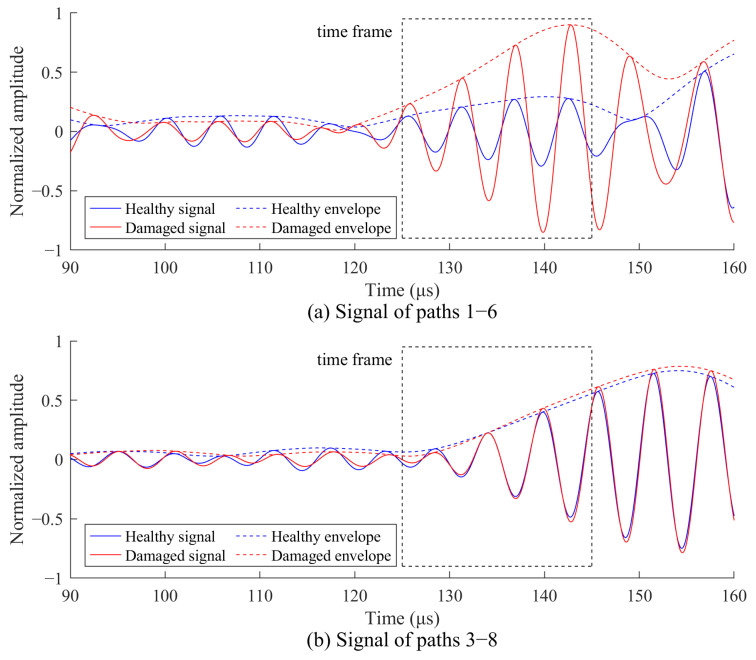
Response signals before and after corrosion in the single corrosion test: (**a**) paths 1–6; (**b**) paths 3–8.

Then, the signals before and after bolt corrosion are analyzed. Figure 12 shows the received Lamb waves along paths 1–6 through the corrosion and paths 3–8, which do not pass through the corrosion. The time frame of the direct A0 wave packet is obtained by calculating the arrival time from the group velocity in Figure 1. In Figure 12a, the signal amplitude within the time frame along path 1–6 shows significant changes, indicating that this path passes through the corrosion region. In Figure 12b, the signal amplitude along path 3–8 remains largely unchanged, indicating that this path does not pass through the corrosion region. The signal amplitude within the time frame is substituted into Equation (8) to calculate the SDC.

**Figure 13:**
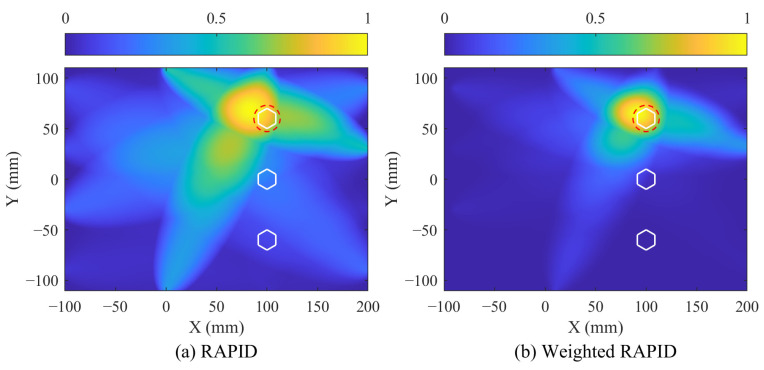
Imaging results of single corrosion through: (**a**) RAPID method, (**b**) Weighted RAPID method. The white hexagons denote the bolts, and the red dotted circle denotes actual corrosion.

Following this, the weighted RAPID imaging is performed using the SDC and the weighting coefficients. Figure 13 illustrates the imaging results for both the traditional RAPID and the weighted RAPID methods for comparison. The bolts are indicated by white hexagons, and the corrosion location is marked by a red dotted circle. Notably, the imaging result of weighted RAPID shows closer agreement with the actual corrosion compared to traditional RAPID. Additionally, Table 3 summarizes the location deviations for corrosion identified by both methods, with the weighted RAPID method achieving the smallest deviation of 6.52 mm. This indicates that the weighted RAPID method offers the highest localization accuracy and superior performance in single corrosion monitoring.

### 4.2. Imaging of the Two Corrosions

**Figure 14:**
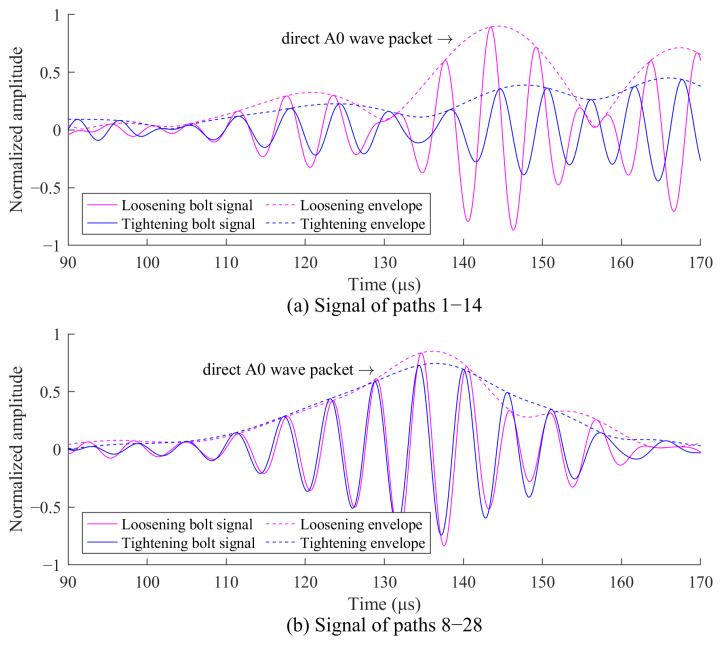
Response signals before and after bolt tightening in the two-corrosion test: (**a**) paths 1–14; (**b**) paths 8–28.

**Figure 15:**
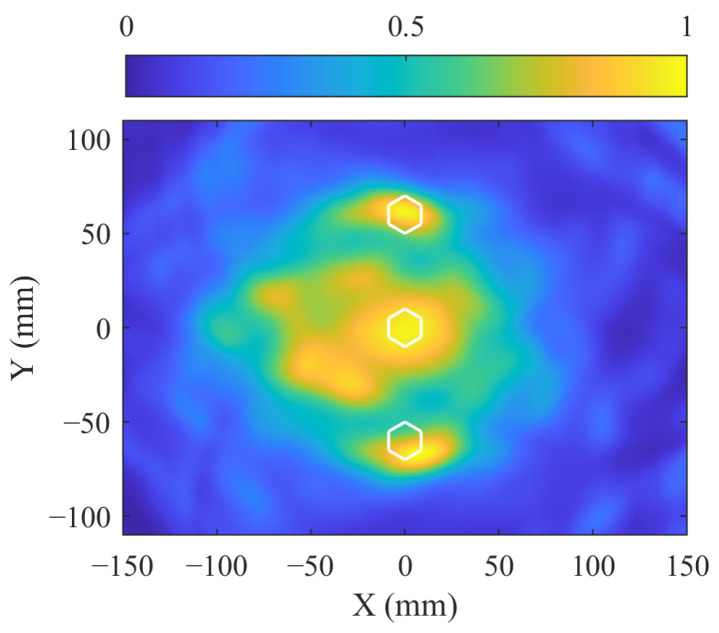
Imaging result of bolt localization using MVDR in the two-corrosion test, with the white hexagons denoting the bolts.

Similar to Section 4.1, bolt imaging is first performed using the signals before and after bolt tightening. Figure 14 shows the received signals along paths 1–14 that pass through the bolts and paths 8–28 that do not pass through the bolts. In Figure 14a, the signal amplitudes of the direct A0 wave packet are influenced by bolt tightening. In Figure 14b, the signal amplitudes of the direct A0 wave packet remain relatively unchanged. These signal characteristics are used for the bolt localization, as shown in Figure 15. The highlighted imaging area in Figure 15 covers three bolts, with 114 sensing paths passing through this region, while 180 sensing paths do not. A weighting coefficient for the 294 sensing paths can then be determined using Equation (9).

**Figure 16:**
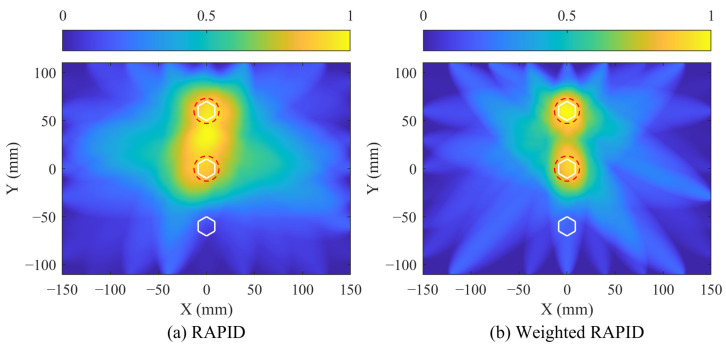
Imaging results of two corrosion test through: (**a**) RAPID method, (**b**) Weighted RAPID method. The white hexagons denote the bolts, and the red dotted circle denotes actual corrosion.

Next, damage imaging is performed using the signals obtained before and after bolt corrosion along with the weighting coefficients. The arrival time and time frame for the direct A0 wave packet can be calculated, and the signal amplitudes within this time frame are substituted into Equation (8) for SDC calculation. Following this, weighted RAPID imaging is performed using the SDC and the weighting coefficients. Figure 16 illustrates the imaging results for both the traditional RAPID and the weighted RAPID methods for comparison. The bolts are indicated by white hexagons, and the corrosion locations are marked by red dotted circles. It can be observed that when *β* = 1.05, the traditional RAPID method characterizes the area between the two corroded bolts as a damaged region. In contrast, the weighted RAPID method avoids this issue and enables accurate localization. The location deviations of the two corrosions identified by the weighted RAPID methods are summarized in Table 4, with a maximum location deviation of 7.43 mm. This demonstrates that the weighted RAPID method offers high localization accuracy in the two-corrosion case, highlighting its effectiveness in identifying multiple corrosions within the bolted lap joint.

## 5. Conclusions

This paper presents a novel corrosion monitoring procedure in bolted lap joints using a Lamb wave-based weighted fusion imaging method. This innovative method integrates the advantages of the MVDR algorithm for identifying bolt loosening and tightening states, along with the RAPID algorithm for damage imaging. Firstly, MVDR is utilized to process Lamb wave signals collected under both bolt-loosening and bolt-tightening conditions to image the bolt locations. Path weighting coefficients are extracted from the imaging results of bolt localization and a weighted RAPID algorithm is proposed, which is more suitable for corrosion monitoring in bolted lap joints. Damage imaging of corroded bolts is performed using Lamb wave signals before and after corrosion. To validate this method, single-corrosion and two-corrosion tests are conducted on three-bolted lap joint specimens with a piezoelectric sensor array. The results demonstrate that the proposed method demonstrated the highest imaging accuracy in comparison with the traditional RAPID algorithm and achieves maximum location deviations of 7.43 mm in two-corrosion imaging. Therefore, the proposed weighted fusion imaging method is more suitable for the visual assessment of multi-damage issues and can be applied to corrosion detection in complex structures such as automobiles, aircraft and ships. The main conclusions drawn from this paper are as follows:

- The weighted fusion imaging method integrates the advantages of the MVDR algorithm for identifying bolt loosening and tightening states, along with the RAPID algorithm for damage imaging. It utilizes the MVDR-based bolt localization results as prior information to weight the sensing paths and employs a weighted RAPID method for accurate damage imaging and localization of corroded bolts.
- The proposed weighted RAPID method enables accurate imaging and localization, outperforming the traditional RAPID method. In single-corrosion imaging, this method achieves a location deviation of 6.52 mm. In two-corrosion imaging, leveraging data from a greater number of sensing paths, this method demonstrates higher localization accuracy with a maximum location deviation of 7.43 mm. These results indicate that the weighted RAPID method is suitable for the visual assessment of multi-damage issues in complex structures.
- To enhance the application potential of the proposed method, future research could focus on investigating the impact of noise on the imaging algorithm by introducing noise into the excitation signals. Additionally, the feasibility of applying this algorithm to other types of sensors and the reliability of long-term monitoring could be explored. The findings of this study are also expected to be integrated with V2X communication technology, enabling the incorporation of the algorithm into automotive structural health monitoring systems for remote damage diagnosis in complex structures.

## Figures and Tables

**Table 1:** Material properties of the aluminum plate.

Material	Young’s Modulus (GPa)	Poisson’s Ratio	Density (kg·m^−3^)
2024-T3 Aluminum alloy	72	0.33	2780

**Table 2:** Performance parameters of piezoelectric sensor.

**Product number**	SMD07T05R412WL
**Material**	SM412
**Geometry**	Diameter: 7 mm, thickness: 0.5 mm
**Resonant frequency**	4.25 MHz ± 5%
**Electrostatic capacitance**	2.5 nF ± 30%
**Test Condition**	25 ± 3 °C 40~70% R.H. (Relative Humidity)

**Table 3:** Location deviations of single corrosion with different imaging methods.

Methods	Traditional RAPID	Weighted RAPID
**Deviations (mm)**	18.56	6.52

**Table 4:** Location deviations of two corrosions with weighted RAPID method.

Location	Bolt I	Bolt II
**Deviations (mm)**	4.5	7.43

## Data Availability

Data will be made available on request.
